# 

**Published:** 1908-05-02

**Authors:** 


### BOOKS RECEIVED.

Sisley's, Ltd.

"The Wife, her Book." By Haydn Brown, L.R.C.P.

J. Wright and Co., Bristol.

"The Medical Annual, 1908."

The Ambrose Co., Ltd.

"Insanity, its Causes and Prevention." By Chas. Williams, L.R.C.P.

The Scientific Press, Ltd.

"A Guide to Sick Nursing in the Tropics." By Andrew Duncan, M.D.

Spottiswoode and Co., Ltd.

"The Medical Register, 1908."
"The Dentists' Register, 1908."

### THE GRADUATES' MEDICAL SCHOOLS.

DIARY FOR WEEK MAY 2 to 9.

### MEDICAL GRADUATES' COLLEGE AND POLYCLINIC, Chenies Street, W.C.

**At 5.15 p.m.**

**May 4,** Sir William Collins, **Some Tendencies in Modern Medicine.**

**May 5,** Dr. Harry Campbell, **Jacksonian Epilepsy.**

**May 6,** Mr. E. Treacher Collins, **Convergent Strabismus**

**May 7,** Sir Malcolm Morris, **Pruritus Ani.**

### THE HOSPITAL FOR SICK CHILDREN, Great Ormond Street, W.C.

**At 4 p.m.**

**May 7,** Dr. A. E. Garrod, **Infantile Scurvy.**

### CHARING CROSS HOSPITAL, W.C.

Post-Graduate Demonstrations.

**At 4 p.m.**

**May 7,** Dr. Eden, **Gynæcological.**

THE HOSPITAL *May* 2, 1908.

*Name* ........................................................................

*Address* ........................................................................

........................................................................

This Coupon must accompany manuscript or contributions intended for The Hospital.

